# Detecting Concentration of Analytes with DETECHIP: A Molecular Sensing Array

**DOI:** 10.4236/jst.2013.33015

**Published:** 2013-09

**Authors:** Hannah Johnke, Gary Batres, Mark Wilson, Andrea E. Holmes, Sharmin Sikich

**Affiliations:** Department of Chemistry, Doane College, Crete, USA

**Keywords:** Colorimetric Arrays, Sensors, Analyte Concentration, RGB Analysis, Drug Detection

## Abstract

DETECHIP^®^ is a detection system made of various sensors that has been shown to detect and discriminate between small molecules of interest, including various illicit and over-the-counter drugs. Previously, detection was normalized to a single concentration of analyte. Now this detection assay can detect concentration differences in analytes via red, green, and blue color value changes and shifts in the UV-Vis spectra of the assay. To determine the concentrations differences, the exposed assays were scanned on a flatbed scanner and the images were analyzed for individual RGB values with a custom macro in ImageJ, an image analysis program. Increasing concentrations of the analyte resulted in greater differences in color values between control and analyte wells. These differences showed a linear relationship to concentration change, some with correlation coefficients greater than 98%. This work expands the capability of DETECHIP to give information about the concentration of analyte when the analyte identity is known.

## 1. Introduction

### 1.1. DETECHIP^®^

**Figure 1:**
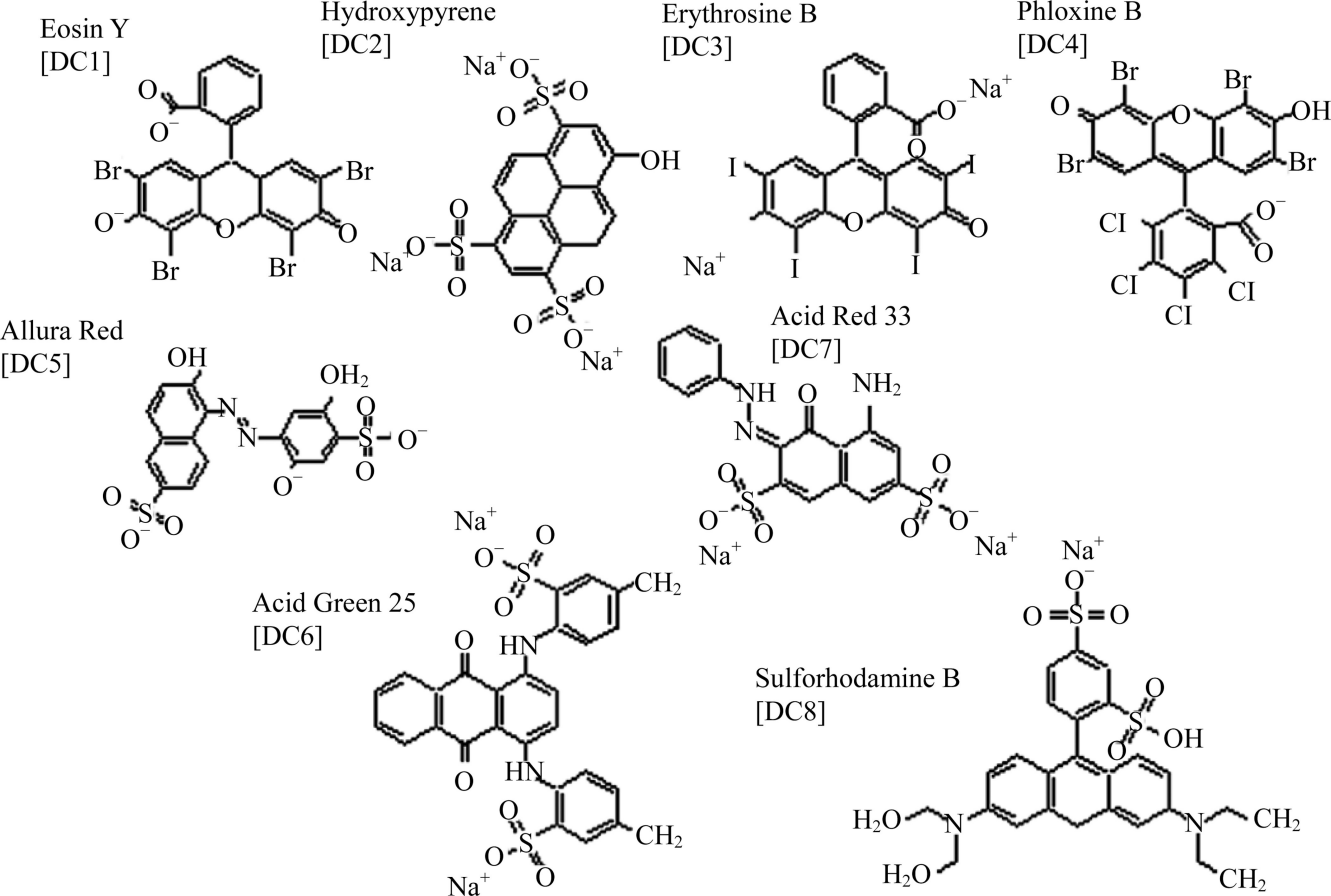
Examples of DETECHIP sensors: Molecular structures of DC1–DC8 and their common chemical names.

DETECHIP^®^, short for detection chip, is a developing technology containing molecular sensors DC1–8 which discriminate between analytes via differential interactions with analytes resulting in colorimetric changes [1]. The molecular structures of molecular sensors DC1–DC8 are shown in Figure 1 [2,3]. This detection technique is a simple assay that has been proven effective in detection of explosives in the field, performance-enhancing drugs in competitive sports, abused narcotics, and other small molecules of interest [4]. Colorimetric changes in DETECHIP are measured with computer analysis of assay images that is able to quantify red, green, and blue (RGB) color values, or by examination of UV-vis spectroscopy of control and analyte-treated solutions [4–6]. Recent work has focused on moving beyond analyte identification and toward analyte concentration determination. In particular, DETECHIP molecular sensors were examined by RGB image analysis and UV-vis spectroscopy to determine if concentration changes can be detected. DETECHIP detection of analyte concentration could provide an alternative to costly, time-consuming methods and expands the capabilities of this detection technique. Thus, it may be possible to apply these quantitative detection assays to applications in forensics, medicine, or homeland security [7–10].

### 1.2. Image Analysis of DETECHIP

**Figure 2:**
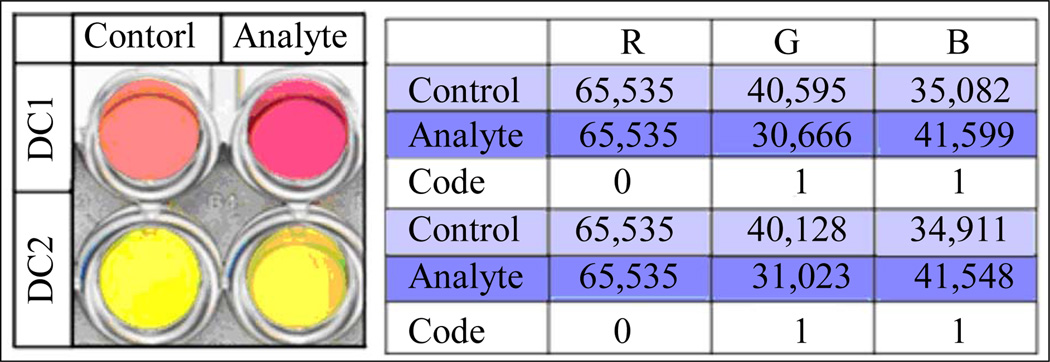
Left—This image shows a visible color change in DC1 but not in DC2. Right—This table shows the resulting code for the given image after RGB analysis. The RGB values in the table represent the total red, green, or blue value for all the pixels in a set area of each well in the image. For DC2, the image analysis detects color change (as indicated by differences in the total color value) in the green and blue channels that the human eye cannot see.

Colorimetric changes exhibited upon addition of analytes to DETECHIP molecular sensors (DC1–DC8) can be detected by analyzing changes in RGB content in an image of the assay. RGB analysis is performed by an in-house modified macro that works with ImageJ (http://imagej.nih.gov/ij/). The macro measures individual red, green, and blue values in an image of a control solution and compares the values to an image of the analyte solution [9,10]. This analysis of DETECHIP has been very successful in determining the identity of analytes [1–3]. As seen in Figure 2, an excerpt of a 96-well DETECHIP plate demonstrates a vivid visible color change in DC1 when the control well and the analyte well are compared. The table in the figure shows three RGB values each for the control and analyte wells. An experimentally determined threshold value of “1000” is used to determine whether the color differences are significant. The red channel is identical for the control and the analyte, whereas the green and blue channels show significant changes between the control and the analyte. Therefore, the red channel gives a code of “0”, whereas the blue and green channels give a code of “1”. Although DC2 does not show a visible color change, computer image analysis finds color changes in the green and blue channels, assigning a value of “1” for both channels. As human vision varies from person to person, the RGB analysis is more objective and less susceptible to human error. Unknown analytes are identified by comparing experimental RGB codes to a previously established library of analyte codes. This master library is updated continuously as more compounds are tested.

DETECHIP with RGB analysis is currently most suited to analysis of compounds at a set concentration and because of this, analytes at alternative concentrations may produce different responses. UV-Vis spectroscopy was also used in conjunction with the image analysis to evaluate if spectroscopic changes in λ_max_ occur when concentrations of analytes are varied. In this study, we show that concentration of analytes can be elucidated through changes in RGB values and with UV-Vis spectroscopy. Ketamine and phenylalanine were selected as the analytes of interest due to their relevance in society. Ketamine has gained much popularity as a recreational drug due to its capability to induce dissociative amnesia [11]. Phenylalanine cannot be metabolized in patients with the genetic disorder phenylketonuria, and the food industry has started to label artificial sweeteners warning consumers of its phenylalanine content [12].

## 2. Materials and Methods

### 2.1. DETECHIP^®^ Plate Preparation

DETECHIP 96-well plates were prepared in a manner similar to previous procedures [1–3].

### 2.2. Analyte Solution Preparation

Reagents used for preparation of the analyte solutions were purchased from Sigma (phenylalanine), and Spectrum Chemicals (ketamine hydrochloride). For RGB analysis, ketamine solutions (CAS #1867-66-9) were prepared in UltraPure water at 10, 25, 50, 62.5, 80, and 100 mM concentrations. DETECHIP plates were then prepared as before, with ketamine added to DETECHIP wells in the same volume but at varying concentrations. Results were analyzed using RGB analysis. Phenylalanine solutions (CAS #150-30-1) were prepared in Ultra-Pure water at concentrations of 20, 40, 60, 80, and 100 mM. Results were analyzed with the same procedure as with ketamine. For UV-Vis analysis, ketamine solutions were prepared in UltraPure water at 5, 10, 20, 30, 45, 60, 80, and 90 mM concentrations.

### 2.3. RGB Analysis

An Epson Perfection V700 photo flatbed scanner was used for RGB analysis. The settings for the scanner were Film (with Film Area Guide) document type, positive film type, 48-bit color, 400 dpi resolution, 8.00 × 10.00 inches document size, and Unsharp Mask on. Images were analyzed using a specialized computer program in ImageJ as previously described [1–3,10]. After much testing, the threshold value of 1000 proved to be optimal for sensitivity and selectivity of most analytes and provided the best and most unique binary codes. If a lower threshold value was selected, too many wells indicated an unreliable color change. Thresholds greater than 1000 did not detect enough color changes. Responses from sensors and RGB codes were examined side by side in order to examine the effect of varying concentration on specific RGB channel. Channels from sensors that displayed a change in code from “0” to “1” as the concentration of ketamine increased were selected. The total color value for that channel in both analyte and control wells was obtained from the macro output, and the difference was calculated by subtracting the specific channel color value of the analyte well from that of the control well. Three plates with three assays each were made, generating nine differences per data point which were averaged and plotted versus ketamine concentration.

### 2.4. UV-Vis Analysis

In order to analyze the spectroscopic changes produced by ketamine interacting with DC1, a DETECHIP assay using only DC1 was prepared in a 96-well plate, with 150 µL of 400 mM phosphate buffer prepared in water (pH 7) and 30 µL of DC1 sensor (750 µM) added to every well. Then 120 mL of analyte solution or water (as the control) was added to each well, diluting the DC1 sensor concentration to 75 µM. Several assays were prepared using varying concentrations of ketamine (described in section 2.2) mixed with DC1 alongside control samples with no ketamine present. The resulting solutions were analyzed using a Cary-50 UV-Vis plate reader.

## 3. Discussion and Results

### 3.1. Concentration Determination through Image Analysis

**Figure 3:**
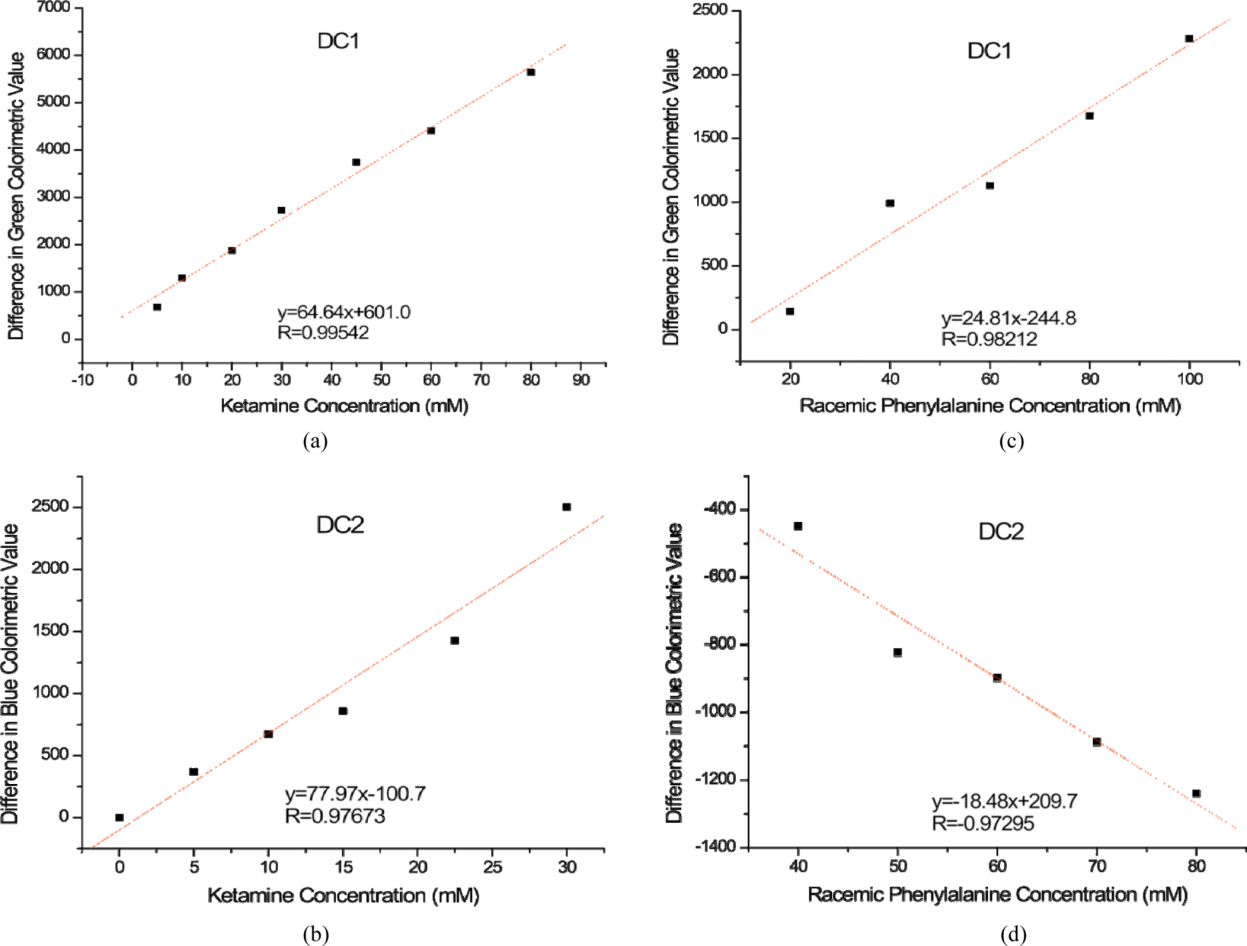
Best linear fit of (a) DC1 and ketamine in the green channel; (b) DC2 and ketamine in the blue channel; (c) DC1 and phenylalanine in the green channel; and (d) DC2 and phenylalanine in the blue channel. All values were calculated by subtracting the green/blue values of the analyte wells from the control wells. The averages of these differences from six trials were then calculated and plotted against concentration of analyte.

For each concentration of ketamine tested from 10 mM to 100 mM, an identifying code was generated as shown in Table 1, with the unique identifying RGB code differing for each concentration. More color changes, or “1” s, develop with increased concentration of ketamine. For example, at 10 mM ketamine, there were 14 color changes observed, and for 25 mM there were 24 color changes. This trend continues until 34 color changes were observed for the highest concentration of 100 mM (Table 1). Data sets for the green (DC1) and blue (DC2) color channels were chosen because a trend in the total color values (either increasing or decreasing compared to control) was noticed with increasing concentration. These data sets were used to calculate average differences between the total color values in analyte-treated and control wells. When the average difference of the green color value in DC1 was plotted against the concentration (Figure 3(a)), a linear relationship between the two parameters occurred with a correlation coefficient of R = 0.99. This could reliably serve as a standard curve for the determination of ketamine concentration. The same trend was observed for ketamine when its concentration was plotted against the difference in blue color values in DC2 (Figure 3(b)). Similar to ketamine, phenylalanine yielded a linear standard curve (R > 0.93) as well when its concentration was plotted against the difference in the green color value in DC1 and blue color value in DC2 (Figures 3(c) and (d)). The red value did not have significant color changes as concentration increased and was not used for the concentration studies of ketamine and phenylalanine (data not shown). Linear relationships were also found in other RGB channels such as the green channel in DC3 with ketamine and the blue channel in DC1 with phenylalanine (data not shown). These results demonstrate that linear standard curves can be obtained for various analytes in order to determine concentration of the analyte tested.

### 3.2. Concentration Determination through UV-Vis Analysis

**Figure 4:**
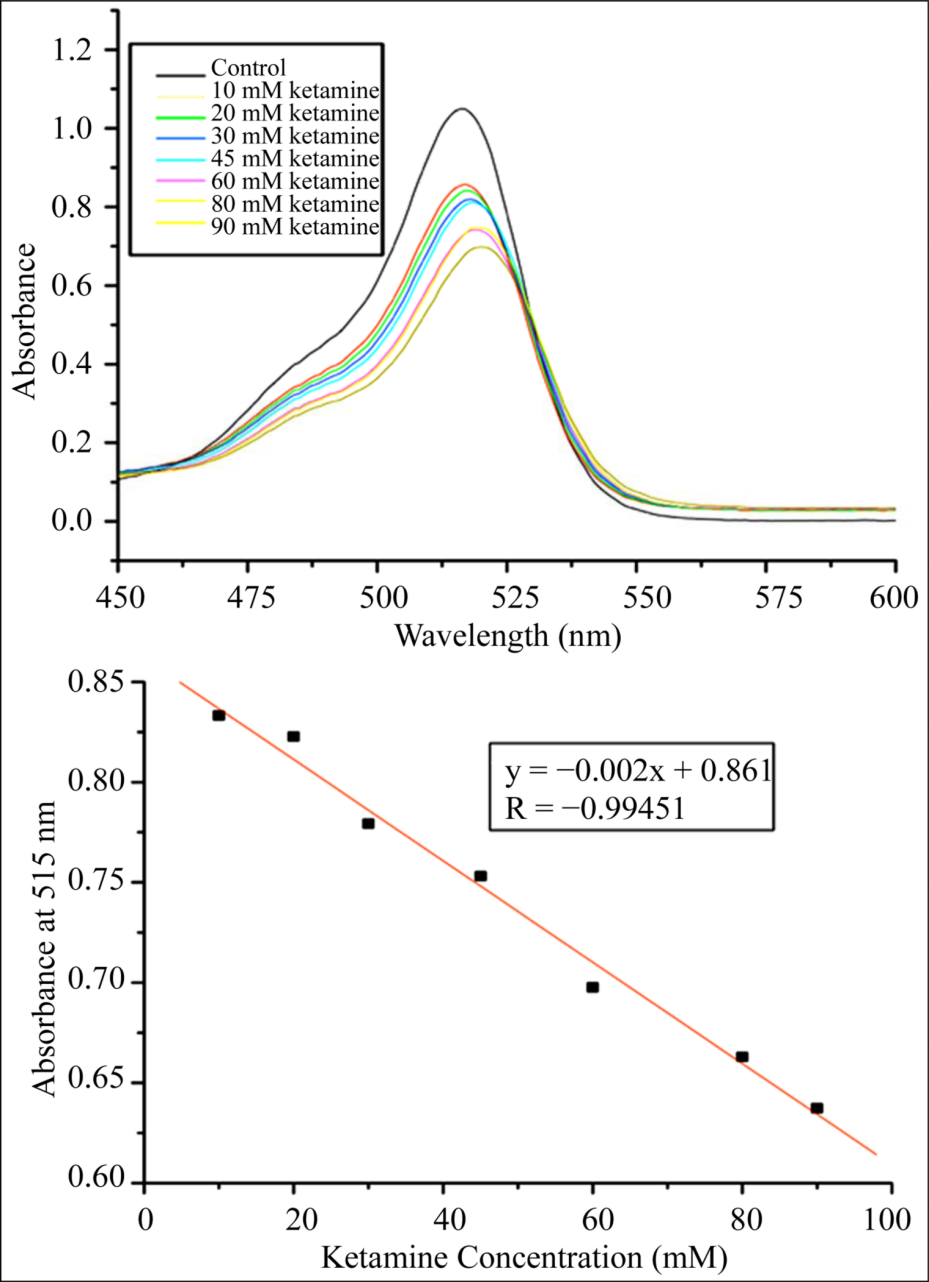
Top—UV-Vis spectra of DC1 with varying concentrations of ketamine, exhibiting a downwards shift in the peak of the spectrum as the concentration of ketamine was increased. Each point on the spectra was calculated from an average of six trials. Bottom—Line of best fit representing the absorbance at 515 nm as ketamine concentration increases.

To complement the results seen in the image analysis, UV-Vis spectra were obtained for solutions with and without the presence of analyte(s) at various concentrations and compared side by side. UV-Vis spectra of ketamine at various concentrations (Figure 4, top) in the presence of DC1 showed two significant results as the concentration of ketamine increased. The maximum absorbance at around 516 nm decreased from A ≈ 1.05 to A ≈ 0.66, a decrease of more than 40%. Also, the maximum wavelength of absorbance at 516 nm for the control shifted 4 nm towards the red region to 520 nm. The spectroscopic changes clearly indicate that there is a strong intermolecular interaction between ketamine and DC1, which becomes more evident as the concentration of ketamine increases. The same trend was observed for phenylalanine, with the maximum wavelength of absorbance shifting approximately 3 nm as the concentration of phenylalanine was increased from 0 mM to 100 mM, and the maximum absorbance decreasing from A ≈ 1.37 to A ≈ 1.28 (results not shown). When the spectroscopic changes, or average absorbance changes, were plotted against the increasing ketamine concentration (Figure 4, bottom), a linear trend was observed (R > 0.98). This linear trend of decreasing absorbance at 515 nm correlates to the linear color change of ketamine in DC1 (Figure 3(a)), confirming our initial hypothesis that colorimetric changes in RGB code are accompanied by spectroscopic changes in absorbance values and shifts of the maximum wavelength.

## 4. Conclusion

In summary, when the identity of the analyte is known, DETECHIP assays can be used to quantify concentration of analytes such as ketamine and phenylalanine. A linear relationship between changing concentration and changing RGB values was found for various DETECHIP sensors (DC1–DC3). A linear relationship in DC1 by UV-Vis spectroscopy was observed between ketamine concentration changes and absorbance changes, indicating that intermolecular interactions (such as proton exchange) of DETECHIP sensors and analytes dictate the color and absorbance changes. Future work will involve analyzing the changing code with concentration to reliably identify unknown analytes, regardless of concentration. Absorbance changes and peak shifts will also be investigated as signatures for identification and concentration determination of analytes. This may lead to a DETECHIP assay that uses multiple, inexpensive techniques for small molecule identification.

## Figures and Tables

**Table 1:** The unique 48-digit codes for DETECHIP with increasing concentrations of ketamine. Additional color changes, highlighted in bold, develop as concentration of ketamine increases, although the concentration of sensor present remains constant. Digits of the code that are exhibited in the graphs in Figure 2 (DC1-green and DC2-blue) are highlighted in yellow, and represent increases or decreases in color change as concentration of ketamine increases. This may result in a change from a “0” to a “1” in the RGB code, if color change is small at lower concentrations and becomes more significant as concentration increases, or can simply be represented by an increase in amount of color change if the code is a “1” for all concentrations.

Ketamine Concentration	48-digit RGB Code	Number of Color Changes
10 mM	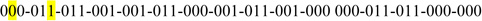	16
25 mM	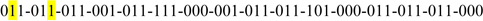	25
50 mM	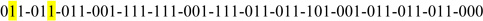	30
62.5 mM	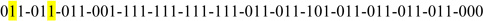	33
80 mM	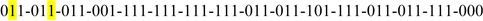	35
100 mM	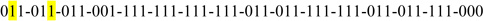	36
